# (*E*)-1-[2-(2-Nitro­styr­yl)-1-phenyl­sulfonyl-1*H*-indol-3-yl]propan-1-one

**DOI:** 10.1107/S1600536814000506

**Published:** 2014-01-15

**Authors:** J. Kanchanadevi, G. Anbalagan, V. Saravanan, A. K. Mohanakrishnan, B. Gunasekaran, V. Manivannan

**Affiliations:** a Department of Physics, Velammal Institute of Technology, Panchetty, Chennai 601 204, India; b Department of Physics, Presidency College (Autonomous), Chennai 600 005, India; c Department of Organic Chemistry, University of Madras, Guindy Campus, Chennai 600 025, India; d Department of Physics & Nano Technology, SRM University, SRM Nagar, Kattankulathur, Kancheepuram Dist, Chennai 603 203, Tamil Nadu, India; e Department of Research and Development, PRIST University, Vallam, Thanjavur 613 403, Tamil Nadu, India

## Abstract

In the title compound, C_25_H_20_N_2_O_5_S, the phenyl ring makes dihedral angles of 89.88 (8) and 13.98 (8)°, respectively, with the indole ring system and the nitro­benzene ring. The dihedral angle between the indole ring system and the nitro­benzene ring is 88.48 (11)°. The mol­ecular structure is stabilized by a weak intra­molecular C—H⋯O inter­action. In the crystal, π–π inter­actions, with centroid–centroid distances of 3.6741 (18) and 3.8873 (17) Å, link the mol­ecules into layers parallel to the *ab* plane.

## Related literature   

For biological activity of indole derivatives, see: Andreani *et al.* (2001 ▶); Quetin-Leclercq (1994 ▶); Mukhopadhyay *et al.* (1981 ▶); Singh *et al.* (2000 ▶). For related structures, see: Umadevi *et al.* (2013 ▶); Kanchanadevi *et al.* (2014 ▶).
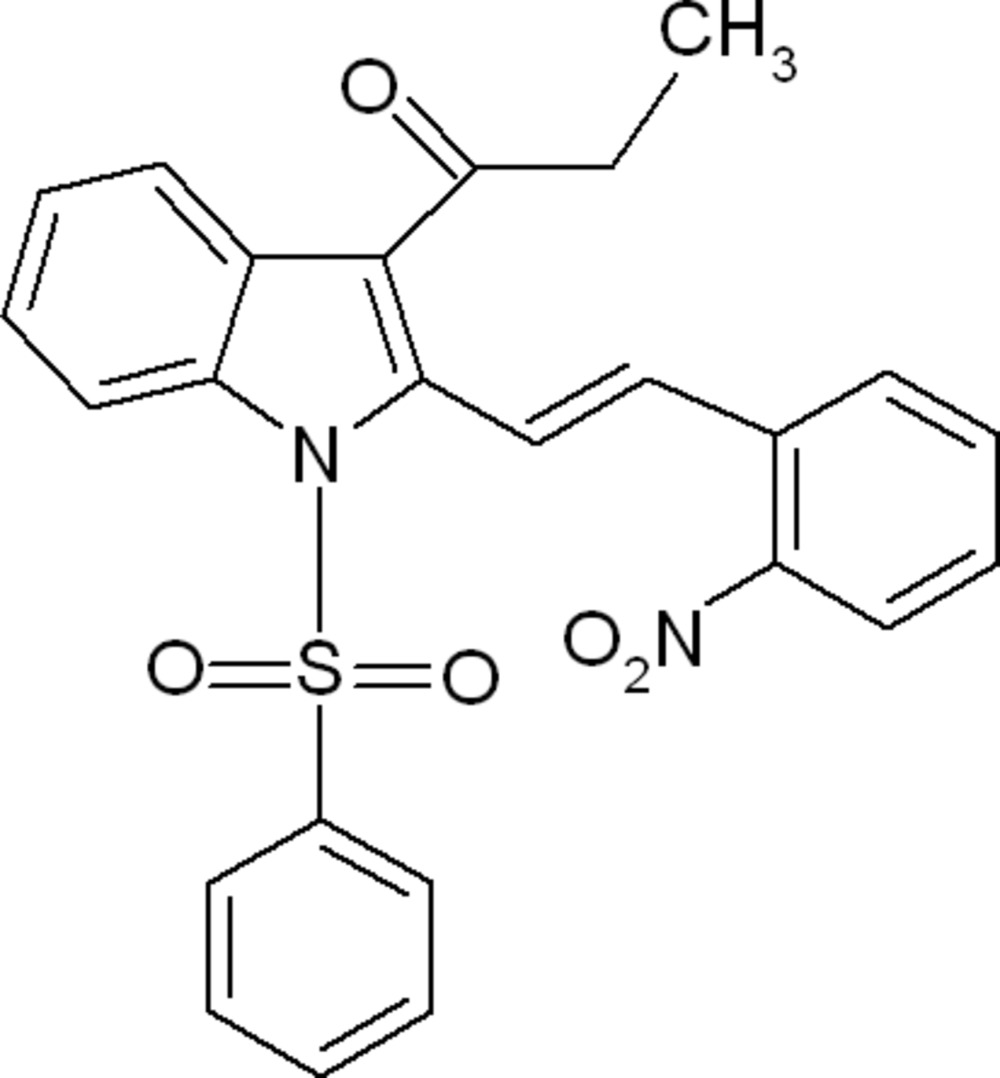


## Experimental   

### 

#### Crystal data  

C_25_H_20_N_2_O_5_S
*M* *_r_* = 460.49
Monoclinic, 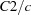

*a* = 21.6100 (18) Å
*b* = 8.3072 (7) Å
*c* = 25.898 (3) Å
β = 112.379 (2)°
*V* = 4299.1 (7) Å^3^
*Z* = 8
Mo *K*α radiation
μ = 0.19 mm^−1^
*T* = 295 K
0.30 × 0.25 × 0.25 mm

#### Data collection  

Bruker APEXII CCD diffractometer
Absorption correction: multi-scan (*SADABS*; Sheldrick, 1996 ▶) *T*_min_ = 0.945, *T*_max_ = 0.955
40633 measured reflections
5343 independent reflections
3700 reflections with *I* > 2σ(*I*)
*R*_int_ = 0.044

#### Refinement  

*R*[*F* ^2^ > 2σ(*F* ^2^)] = 0.051
*wR*(*F* ^2^) = 0.151
*S* = 1.04
5343 reflections
299 parameters
H-atom parameters constrained
Δρ_max_ = 0.66 e Å^−3^
Δρ_min_ = −0.67 e Å^−3^

### 

Data collection: *APEX2* (Bruker, 2008 ▶); cell refinement: *SAINT* (Bruker, 2008 ▶); data reduction: *SAINT*; program(s) used to solve structure: *SHELXS97* (Sheldrick, 2008 ▶); program(s) used to refine structure: *SHELXL97* (Sheldrick, 2008 ▶); molecular graphics: *PLATON* (Spek, 2009 ▶); software used to prepare material for publication: *SHELXL97*.

## Supplementary Material

Crystal structure: contains datablock(s) I. DOI: 10.1107/S1600536814000506/is5331sup1.cif

Structure factors: contains datablock(s) I. DOI: 10.1107/S1600536814000506/is5331Isup2.hkl

Supporting information file. DOI: 10.1107/S1600536814000506/is5331Isup3.cml

Additional supporting information:  crystallographic information; 3D view; checkCIF report

## Figures and Tables

**Table 1:** Hydrogen-bond geometry (Å, °)

*D*—H⋯*A*	*D*—H	H⋯*A*	*D*⋯*A*	*D*—H⋯*A*
C2—H2⋯O1	0.93	2.32	2.912 (3)	121

**Fig. 1.**
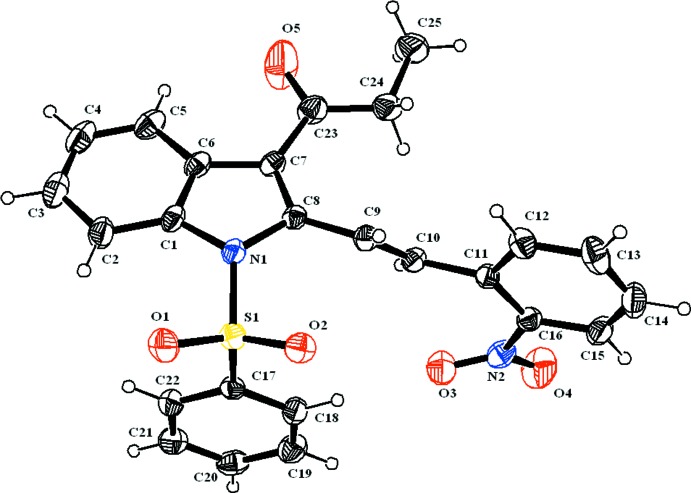
The molecular structure of the title compound, with atom labels and 30% probability displacement ellipsoids for non-H atoms.

## supplementary crystallographic information

### 1. Comment

Indole derivatives exhibits antibacterial, antifungal (Singh *et al.*, 2000) and antitumour activities (Andreani *et al.*, 2001). Some of the indole alkaloids extracted from plants possess interesting cytotoxic and antiparasitic properties (Quetin-Leclercq, 1994; Mukhopadhyay *et al.*, 1981).

The geometric parameters of the title molecule (Fig. 1) agree well with reported similar structure (Umadevi *et al.*, 2013). The phenyl ring makes a dihedral angles of 89.88 (8) and 13.98 (8)° with the indole ring system and the nitro benzene ring, respectively. The sum of bond angles around the atom N1 [356.03°] indicates *sp^2^* hybridized. The molecular structure is stabilized by a weak intramolecular C—H···O interaction.

### 2. Experimental

A solution of 1-[2-(bromomethyl)-1-(phenylsulfonyl)-1*H*-indol-3-yl] propan-1-one (5 g, 12.31 mmol) and triphenylphosphine (3.5 g, 13.54 mmol) in dry THF (100 ml) was refluxed for 6 h. After consumption of the starting material, the solvent was removed under vaccum and the solid was washed with diethyl ether to give the phosphonium salt. Then, the mixture of phosphonium salt (8 g, 11.97 mmol), 2-nitrobenzaldehydes (1.99 g, 13.17 mmol) and K_2_CO_3_ (3.30 g, 23.95 mmol) in DCM (70 ml) was stirred at room temperature for 24 h. After completion of the reaction (monitored by TLC), it was diluted using DCM (30 ml), washed with water (2 × 100 ml) and dried (Na_2_SO_4_). Removal of solvent followed by trituration of the crude product with MeOH (20 ml) afforded (*E*)-1-[2-(2-nitrostyryl)-1-(phenylsulfonyl)-1*H*- indol-3-yl]propan-1-one as yellow solid (4.30 g, 76%) with melting point 168–170 °C.

### 3. Refinement

H atoms were positioned geometrically and refined using riding model with C—H = 0.93 Å and *U*_iso_(H) = 1.2*U*_eq_(C) for aromatic CH, C—H = 0.97 Å and *U*_iso_(H) = 1.2*U*_eq_(C) for CH_2_, and C—H = 0.96 Å and *U*_iso_(H) = 1.5*U*_eq_(C) for CH_3_.

### Crystal data

**Table:**

C_25_H_20_N_2_O_5_S	*F*(000) = 1920
*M**_r_* = 460.49	*D*_x_ = 1.423 Mg m^−^^3^
Monoclinic, *C*2/*c*	Mo *K*α radiation, λ = 0.71073 Å
Hall symbol: -C 2yc	Cell parameters from 5350 reflections
*a* = 21.6100 (18) Å	θ = 2.0–28.3°
*b* = 8.3072 (7) Å	µ = 0.19 mm^−^^1^
*c* = 25.898 (3) Å	*T* = 295 K
β = 112.379 (2)°	Block, yellow
*V* = 4299.1 (7) Å^3^	0.30 × 0.25 × 0.25 mm
*Z* = 8	

### Data collection

**Table:**

Bruker APEXII CCD diffractometer	5343 independent reflections
Radiation source: fine-focus sealed tube	3700 reflections with *I* > 2σ(*I*)
Graphite monochromator	*R*_int_ = 0.044
Detector resolution: 0 pixels mm^-1^	θ_max_ = 28.3°, θ_min_ = 2.0°
ω and φ scans	*h* = −28→28
Absorption correction: multi-scan (*SADABS*; Sheldrick, 1996)	*k* = −11→11
*T*_min_ = 0.945, *T*_max_ = 0.955	*l* = −32→34
40633 measured reflections	

### Refinement

**Table:**

Refinement on *F*^2^	Primary atom site location: structure-invariant direct methods
Least-squares matrix: full	Secondary atom site location: difference Fourier map
*R*[*F*^2^ > 2σ(*F*^2^)] = 0.051	Hydrogen site location: inferred from neighbouring sites
*wR*(*F*^2^) = 0.151	H-atom parameters constrained
*S* = 1.04	*w* = 1/[σ^2^(*F*_o_^2^) + (0.0587*P*)^2^ + 6.0066*P*] where *P* = (*F*_o_^2^ + 2*F*_c_^2^)/3
5343 reflections	(Δ/σ)_max_ = 0.001
299 parameters	Δρ_max_ = 0.66 e Å^−^^3^
0 restraints	Δρ_min_ = −0.67 e Å^−^^3^

### Fractional atomic coordinates and isotropic or equivalent isotropic displacement parameters (Å^2^)

**Table:**

	*x*	*y*	*z*	*U*_iso_*/*U*_eq_	
C1	0.15190 (11)	−0.0496 (3)	0.50378 (9)	0.0411 (5)	
C2	0.08840 (13)	−0.1098 (3)	0.47316 (10)	0.0544 (7)	
H2	0.0544	−0.1071	0.4869	0.065*	
C3	0.07820 (16)	−0.1738 (3)	0.42139 (11)	0.0649 (8)	
H3	0.0364	−0.2157	0.3997	0.078*	
C4	0.12856 (17)	−0.1769 (3)	0.40107 (11)	0.0647 (8)	
H4	0.1199	−0.2207	0.3659	0.078*	
C5	0.19136 (15)	−0.1172 (3)	0.43131 (10)	0.0561 (7)	
H5	0.2249	−0.1208	0.4171	0.067*	
C6	0.20352 (12)	−0.0506 (3)	0.48417 (9)	0.0433 (5)	
C7	0.26149 (11)	0.0260 (3)	0.52583 (9)	0.0400 (5)	
C8	0.24458 (10)	0.0690 (3)	0.56984 (8)	0.0356 (5)	
C9	0.28201 (11)	0.1582 (3)	0.62103 (9)	0.0384 (5)	
H9	0.2654	0.2575	0.6262	0.046*	
C10	0.33832 (11)	0.1058 (3)	0.66058 (8)	0.0384 (5)	
H10	0.3533	0.0032	0.6568	0.046*	
C11	0.37820 (10)	0.2010 (3)	0.71009 (9)	0.0374 (5)	
C12	0.37603 (13)	0.3685 (3)	0.70753 (11)	0.0503 (6)	
H12	0.3482	0.4178	0.6746	0.060*	
C13	0.41344 (15)	0.4635 (4)	0.75177 (14)	0.0651 (8)	
H13	0.4098	0.5749	0.7487	0.078*	
C14	0.45614 (14)	0.3952 (4)	0.80052 (12)	0.0661 (8)	
H14	0.4814	0.4598	0.8304	0.079*	
C15	0.46118 (13)	0.2320 (4)	0.80475 (10)	0.0566 (7)	
H15	0.4906	0.1847	0.8374	0.068*	
C16	0.42243 (11)	0.1363 (3)	0.76039 (9)	0.0415 (5)	
C17	0.16595 (11)	−0.1823 (3)	0.63322 (9)	0.0372 (5)	
C18	0.22218 (12)	−0.1958 (3)	0.68196 (10)	0.0479 (6)	
H18	0.2470	−0.1051	0.6987	0.057*	
C19	0.24071 (14)	−0.3460 (3)	0.70527 (11)	0.0578 (7)	
H19	0.2782	−0.3571	0.7381	0.069*	
C20	0.20406 (15)	−0.4794 (3)	0.68021 (11)	0.0549 (7)	
H20	0.2169	−0.5802	0.6964	0.066*	
C21	0.14871 (15)	−0.4656 (3)	0.63161 (11)	0.0548 (7)	
H21	0.1243	−0.5567	0.6149	0.066*	
C22	0.12933 (12)	−0.3159 (3)	0.60758 (10)	0.0473 (6)	
H22	0.0920	−0.3055	0.5745	0.057*	
C23	0.32263 (14)	0.0616 (4)	0.51526 (11)	0.0565 (7)	
C25	0.43883 (15)	0.1576 (5)	0.53443 (13)	0.0788 (10)	
H25A	0.4556	0.0538	0.5299	0.118*	
H25B	0.4744	0.2201	0.5605	0.118*	
H25C	0.4216	0.2120	0.4991	0.118*	
N1	0.17663 (9)	0.0287 (2)	0.55666 (7)	0.0387 (4)	
N2	0.42953 (12)	−0.0372 (3)	0.76957 (9)	0.0551 (6)	
O1	0.07086 (8)	0.0081 (2)	0.57260 (8)	0.0582 (5)	
O2	0.16992 (9)	0.1277 (2)	0.64547 (7)	0.0495 (4)	
O3	0.38239 (11)	−0.1241 (2)	0.74363 (8)	0.0685 (6)	
O4	0.48249 (12)	−0.0875 (3)	0.80380 (10)	0.0950 (8)	
O5	0.32135 (15)	0.0336 (6)	0.46959 (12)	0.1606 (19)	
C24	0.38311 (13)	0.1372 (4)	0.55629 (11)	0.0596 (7)	
H24A	0.3994	0.0716	0.5898	0.072*	
H24B	0.3714	0.2420	0.5664	0.072*	
S1	0.14097 (3)	0.00862 (7)	0.60389 (2)	0.04073 (16)	

### Atomic displacement parameters (Å^2^)

**Table:**

	*U*^11^	*U*^22^	*U*^33^	*U*^12^	*U*^13^	*U*^23^
C1	0.0433 (12)	0.0396 (12)	0.0310 (10)	0.0040 (9)	0.0034 (9)	0.0030 (9)
C2	0.0478 (14)	0.0557 (15)	0.0461 (13)	−0.0036 (12)	0.0025 (11)	0.0019 (12)
C3	0.0667 (18)	0.0546 (16)	0.0486 (15)	−0.0027 (14)	−0.0058 (14)	−0.0016 (13)
C4	0.089 (2)	0.0498 (15)	0.0366 (13)	0.0053 (15)	0.0028 (14)	−0.0063 (11)
C5	0.0748 (18)	0.0494 (14)	0.0405 (13)	0.0120 (13)	0.0180 (13)	−0.0017 (11)
C6	0.0512 (13)	0.0389 (12)	0.0339 (11)	0.0084 (10)	0.0095 (10)	0.0037 (9)
C7	0.0407 (12)	0.0423 (12)	0.0352 (11)	0.0064 (9)	0.0123 (9)	0.0031 (9)
C8	0.0356 (11)	0.0343 (10)	0.0332 (10)	0.0040 (8)	0.0090 (8)	0.0050 (8)
C9	0.0389 (11)	0.0376 (11)	0.0385 (11)	0.0001 (9)	0.0145 (9)	−0.0019 (9)
C10	0.0428 (12)	0.0363 (11)	0.0347 (10)	0.0010 (9)	0.0131 (9)	0.0001 (9)
C11	0.0344 (10)	0.0423 (12)	0.0367 (11)	−0.0020 (9)	0.0147 (9)	−0.0035 (9)
C12	0.0466 (13)	0.0431 (13)	0.0569 (15)	−0.0023 (11)	0.0149 (11)	−0.0004 (11)
C13	0.0616 (18)	0.0473 (15)	0.086 (2)	−0.0141 (13)	0.0275 (16)	−0.0191 (14)
C14	0.0574 (17)	0.078 (2)	0.0618 (17)	−0.0217 (15)	0.0214 (14)	−0.0309 (16)
C15	0.0452 (14)	0.085 (2)	0.0363 (12)	−0.0074 (13)	0.0113 (11)	−0.0076 (13)
C16	0.0363 (11)	0.0537 (14)	0.0349 (11)	−0.0011 (10)	0.0141 (9)	−0.0005 (10)
C17	0.0365 (11)	0.0407 (11)	0.0379 (11)	−0.0014 (9)	0.0182 (9)	−0.0015 (9)
C18	0.0489 (13)	0.0472 (13)	0.0415 (12)	−0.0043 (11)	0.0105 (11)	0.0003 (10)
C19	0.0615 (16)	0.0576 (16)	0.0476 (14)	0.0064 (13)	0.0131 (12)	0.0084 (12)
C20	0.0778 (19)	0.0422 (13)	0.0542 (15)	0.0059 (13)	0.0358 (14)	0.0061 (11)
C21	0.0716 (18)	0.0438 (14)	0.0562 (15)	−0.0095 (12)	0.0324 (14)	−0.0099 (11)
C22	0.0475 (13)	0.0507 (14)	0.0432 (13)	−0.0071 (11)	0.0168 (11)	−0.0063 (11)
C23	0.0539 (15)	0.0727 (18)	0.0490 (14)	0.0045 (13)	0.0263 (12)	−0.0045 (13)
C25	0.0535 (17)	0.117 (3)	0.071 (2)	−0.0062 (18)	0.0306 (15)	0.0107 (19)
N1	0.0353 (9)	0.0444 (10)	0.0335 (9)	−0.0002 (8)	0.0096 (7)	0.0003 (8)
N2	0.0550 (13)	0.0605 (14)	0.0427 (11)	0.0093 (11)	0.0106 (10)	0.0101 (10)
O1	0.0334 (9)	0.0724 (12)	0.0652 (11)	0.0094 (8)	0.0146 (8)	0.0086 (10)
O2	0.0594 (10)	0.0413 (9)	0.0533 (10)	0.0017 (8)	0.0276 (8)	−0.0066 (7)
O3	0.0716 (13)	0.0500 (11)	0.0676 (12)	−0.0019 (10)	0.0083 (10)	0.0098 (10)
O4	0.0792 (15)	0.0911 (17)	0.0785 (15)	0.0235 (13)	−0.0106 (12)	0.0267 (13)
O5	0.099 (2)	0.318 (5)	0.0942 (19)	−0.081 (3)	0.0701 (17)	−0.103 (3)
C24	0.0500 (15)	0.0769 (19)	0.0548 (15)	−0.0035 (13)	0.0232 (12)	0.0066 (14)
S1	0.0360 (3)	0.0430 (3)	0.0434 (3)	0.0043 (2)	0.0154 (2)	0.0012 (2)

### Geometric parameters (Å, º)

**Table:**

C1—C6	1.390 (3)	C15—C16	1.386 (3)
C1—C2	1.390 (3)	C15—H15	0.9300
C1—N1	1.424 (3)	C16—N2	1.460 (3)
C2—C3	1.380 (4)	C17—C22	1.378 (3)
C2—H2	0.9300	C17—C18	1.384 (3)
C3—C4	1.378 (5)	C17—S1	1.753 (2)
C3—H3	0.9300	C18—C19	1.378 (4)
C4—C5	1.376 (4)	C18—H18	0.9300
C4—H4	0.9300	C19—C20	1.373 (4)
C5—C6	1.405 (3)	C19—H19	0.9300
C5—H5	0.9300	C20—C21	1.372 (4)
C6—C7	1.452 (3)	C20—H20	0.9300
C7—C8	1.370 (3)	C21—C22	1.382 (4)
C7—C23	1.478 (4)	C21—H21	0.9300
C8—N1	1.415 (3)	C22—H22	0.9300
C8—C9	1.464 (3)	C23—O5	1.195 (3)
C9—C10	1.331 (3)	C23—C24	1.475 (4)
C9—H9	0.9300	C25—C24	1.523 (4)
C10—C11	1.474 (3)	C25—H25A	0.9600
C10—H10	0.9300	C25—H25B	0.9600
C11—C12	1.393 (3)	C25—H25C	0.9600
C11—C16	1.397 (3)	N1—S1	1.6838 (19)
C12—C13	1.372 (4)	N2—O3	1.220 (3)
C12—H12	0.9300	N2—O4	1.224 (3)
C13—C14	1.371 (4)	O1—S1	1.4204 (17)
C13—H13	0.9300	O2—S1	1.4198 (17)
C14—C15	1.361 (4)	C24—H24A	0.9700
C14—H14	0.9300	C24—H24B	0.9700
			
C6—C1—C2	123.0 (2)	C11—C16—N2	121.6 (2)
C6—C1—N1	107.12 (19)	C22—C17—C18	121.2 (2)
C2—C1—N1	129.8 (2)	C22—C17—S1	119.64 (17)
C3—C2—C1	116.8 (3)	C18—C17—S1	119.14 (17)
C3—C2—H2	121.6	C19—C18—C17	118.7 (2)
C1—C2—H2	121.6	C19—C18—H18	120.6
C4—C3—C2	121.4 (3)	C17—C18—H18	120.6
C4—C3—H3	119.3	C20—C19—C18	120.3 (2)
C2—C3—H3	119.3	C20—C19—H19	119.8
C5—C4—C3	121.8 (3)	C18—C19—H19	119.8
C5—C4—H4	119.1	C21—C20—C19	120.7 (2)
C3—C4—H4	119.1	C21—C20—H20	119.6
C4—C5—C6	118.3 (3)	C19—C20—H20	119.6
C4—C5—H5	120.8	C20—C21—C22	119.8 (2)
C6—C5—H5	120.8	C20—C21—H21	120.1
C1—C6—C5	118.6 (2)	C22—C21—H21	120.1
C1—C6—C7	108.1 (2)	C17—C22—C21	119.2 (2)
C5—C6—C7	133.2 (2)	C17—C22—H22	120.4
C8—C7—C6	107.7 (2)	C21—C22—H22	120.4
C8—C7—C23	130.0 (2)	O5—C23—C24	118.4 (3)
C6—C7—C23	122.0 (2)	O5—C23—C7	117.3 (3)
C7—C8—N1	108.57 (18)	C24—C23—C7	124.2 (2)
C7—C8—C9	130.9 (2)	C24—C25—H25A	109.5
N1—C8—C9	120.19 (19)	C24—C25—H25B	109.5
C10—C9—C8	124.0 (2)	H25A—C25—H25B	109.5
C10—C9—H9	118.0	C24—C25—H25C	109.5
C8—C9—H9	118.0	H25A—C25—H25C	109.5
C9—C10—C11	123.7 (2)	H25B—C25—H25C	109.5
C9—C10—H10	118.2	C8—N1—C1	108.36 (18)
C11—C10—H10	118.2	C8—N1—S1	124.61 (14)
C12—C11—C16	115.3 (2)	C1—N1—S1	123.06 (16)
C12—C11—C10	119.8 (2)	O3—N2—O4	123.5 (3)
C16—C11—C10	124.8 (2)	O3—N2—C16	118.7 (2)
C13—C12—C11	122.4 (3)	O4—N2—C16	117.8 (2)
C13—C12—H12	118.8	C23—C24—C25	112.9 (2)
C11—C12—H12	118.8	C23—C24—H24A	109.0
C14—C13—C12	120.4 (3)	C25—C24—H24A	109.0
C14—C13—H13	119.8	C23—C24—H24B	109.0
C12—C13—H13	119.8	C25—C24—H24B	109.0
C15—C14—C13	119.4 (3)	H24A—C24—H24B	107.8
C15—C14—H14	120.3	O2—S1—O1	120.06 (11)
C13—C14—H14	120.3	O2—S1—N1	106.89 (10)
C14—C15—C16	120.0 (3)	O1—S1—N1	105.65 (10)
C14—C15—H15	120.0	O2—S1—C17	109.13 (10)
C16—C15—H15	120.0	O1—S1—C17	109.28 (11)
C15—C16—C11	122.4 (2)	N1—S1—C17	104.71 (10)
C15—C16—N2	116.0 (2)		
			
C6—C1—C2—C3	0.6 (4)	C17—C18—C19—C20	0.4 (4)
N1—C1—C2—C3	177.1 (2)	C18—C19—C20—C21	0.2 (4)
C1—C2—C3—C4	−0.3 (4)	C19—C20—C21—C22	−0.2 (4)
C2—C3—C4—C5	0.2 (4)	C18—C17—C22—C21	1.0 (4)
C3—C4—C5—C6	−0.4 (4)	S1—C17—C22—C21	−178.78 (19)
C2—C1—C6—C5	−0.8 (4)	C20—C21—C22—C17	−0.4 (4)
N1—C1—C6—C5	−178.0 (2)	C8—C7—C23—O5	−167.9 (4)
C2—C1—C6—C7	178.1 (2)	C6—C7—C23—O5	4.3 (5)
N1—C1—C6—C7	0.9 (2)	C8—C7—C23—C24	8.5 (4)
C4—C5—C6—C1	0.7 (4)	C6—C7—C23—C24	−179.2 (3)
C4—C5—C6—C7	−177.9 (2)	C7—C8—N1—C1	3.1 (2)
C1—C6—C7—C8	1.0 (3)	C9—C8—N1—C1	177.08 (18)
C5—C6—C7—C8	179.7 (2)	C7—C8—N1—S1	161.12 (16)
C1—C6—C7—C23	−172.8 (2)	C9—C8—N1—S1	−24.9 (3)
C5—C6—C7—C23	5.9 (4)	C6—C1—N1—C8	−2.4 (2)
C6—C7—C8—N1	−2.5 (2)	C2—C1—N1—C8	−179.3 (2)
C23—C7—C8—N1	170.6 (2)	C6—C1—N1—S1	−160.86 (16)
C6—C7—C8—C9	−175.6 (2)	C2—C1—N1—S1	22.2 (3)
C23—C7—C8—C9	−2.5 (4)	C15—C16—N2—O3	152.5 (2)
C7—C8—C9—C10	−64.8 (3)	C11—C16—N2—O3	−26.4 (4)
N1—C8—C9—C10	122.7 (2)	C15—C16—N2—O4	−26.3 (4)
C8—C9—C10—C11	175.6 (2)	C11—C16—N2—O4	154.8 (2)
C9—C10—C11—C12	−27.0 (3)	O5—C23—C24—C25	−4.3 (5)
C9—C10—C11—C16	156.7 (2)	C7—C23—C24—C25	179.3 (3)
C16—C11—C12—C13	−1.6 (4)	C8—N1—S1—O2	36.9 (2)
C10—C11—C12—C13	−178.2 (2)	C1—N1—S1—O2	−168.17 (17)
C11—C12—C13—C14	1.4 (4)	C8—N1—S1—O1	165.85 (18)
C12—C13—C14—C15	0.0 (5)	C1—N1—S1—O1	−39.2 (2)
C13—C14—C15—C16	−1.2 (4)	C8—N1—S1—C17	−78.81 (19)
C14—C15—C16—C11	1.1 (4)	C1—N1—S1—C17	76.13 (19)
C14—C15—C16—N2	−177.9 (2)	C22—C17—S1—O2	159.83 (18)
C12—C11—C16—C15	0.3 (3)	C18—C17—S1—O2	−20.0 (2)
C10—C11—C16—C15	176.8 (2)	C22—C17—S1—O1	26.7 (2)
C12—C11—C16—N2	179.2 (2)	C18—C17—S1—O1	−153.07 (19)
C10—C11—C16—N2	−4.4 (3)	C22—C17—S1—N1	−86.0 (2)
C22—C17—C18—C19	−1.0 (4)	C18—C17—S1—N1	94.2 (2)
S1—C17—C18—C19	178.8 (2)		

### Hydrogen-bond geometry (Å, º)

**Table:**

*D*—H···*A*	*D*—H	H···*A*	*D*···*A*	*D*—H···*A*
C2—H2···O1	0.93	2.32	2.912 (3)	121
